# Glycyrrhizin Ameliorate Ischemia Reperfusion Lung Injury through Downregulate TLR2 Signaling Cascade in Alveolar Macrophages

**DOI:** 10.3389/fphar.2017.00389

**Published:** 2017-06-16

**Authors:** Lin Fei, Feng Jifeng, Wang Tiantian, He Yi, Pan Linghui

**Affiliations:** ^1^Department of Anesthesiology, Affiliated Tumor Hospital of Guangxi Medical UniversityNanning, China; ^2^Department of Anesthesiology, Guangxi Maternal and Child Health HospitalNanning, China

**Keywords:** ischemia reperfusion lung injury, Glycyrrhizin, TLR2, alveolar macrophages, inflammatory mediators

## Abstract

This experiment was conducted to study whether pretreatment with Glycyrrhizin (GL) could ameliorate ischemia-reperfusion (I/R) lung injury and investigate the mechanisms of its protective effects in a mice model. Six-eight weeks male BALB/C mice were randomly assigned to four groups (*n* = 6): Control, Glycyrrhizin, I/R and I/R+Glycyrrhizin. Lung I/R was achieved by clamping the left hilus pulmonis. GL (200 mg/kg) was injected intraperitoneally 30 min before anesthesia. Measurement of pathohistological lung injury score, pulmonary permeability, isolated alveolar macrophages, inflammatory mediators, TLR2 and its downstream factors (MyD88, NF-κB) were performed. The results were as anticipated. Pathohistological evaluation indicated that GL significantly ameliorated I/R-induced lung injury, pulmonary permeability and edema. Pretreatment with GL significantly inhibited I/R-induced inflammation in lung tissues and BALF. In addition, GL significantly decreased I/R-induced isolated alveolar macrophages and suppressed I/R-induced expression of TLR2 and its downstream factors in lung tissues and alveolar macrophages. Collectively, our data indicated that pretreatment with GL could ameliorate I/R lung injury. The mechanisms of its protective effects might be inhibit I/R-induced inflammatory response through downregulate TLR2 signaling cascade in alveolar macrophages.

## Introduction

Lung ischemia-reperfusion injury (LIRI) can occur in lung transplantation, lung resection, cardiac arrest, and pulmonary emboli. Lung dysfunction caused by LIRI is a strong risk factor for acute lung injury (ALI), and portends worse patient outcomes (Fiser et al., 2002; de Perrot et al., 2003; Weyker et al., 2013). Inflammatory response has significant role in tissue damage after organ ischemia-reperfusion (I/R) (Gelderblom et al., 2012; Diamond et al., 2013; Phelan et al., 2015). Recent studies suggest that I/R injury could lead to sterile inflammation through activating the innate immune system. Characterization of the cell-specific sterile inflammatory events in LIRI is a key step toward treatment strategies aimed at alleviating LIRI and improving patient outcomes (Lin et al., 2011; Prakash et al., 2015; Salameh and Dhein, 2015).

Toll-like receptors (TLRs) represent an conserved family of pattern recognition receptors. TLRs in alveolar macrophages have ability to recognize endogenous ligands and play a pivotal role in innate immunity and sterile inflammation. The ability to initiate inflammatory response makes them suitable candidates for early signaling in LIRI (Miyake, 2007; Lin et al., 2011; Zheng et al., 2013). TLR2 has been implicated as a crucial modulator in I/R models and has an important role in the initiation of inflammation during I/R process. Using deletion or pharmacologic antagonism of TLR2 can reduce injury severity in lung, renal, and cerebral models of ischemia reperfusion (Favre et al., 2007; Shigeoka et al., 2007; Phelan et al., 2015). TLR2 signal is required for recruiting Myeloid differentiation factor 88 (MyD88) to the receptors. These kinases ultimately activate transcription factors such as nuclear factor-κB (NF-κB) which result in production of various proinflammatory cytokines. However, the cellular and molecular pathways responsible for sterile inflammatory events in LIRI have not been fully delineated. How to control the activation of TLR2 signaling pathway in process of I/R lung injury is an important issue to exploration.

Glycyrrhizin (GL), a triterpenoid saponin compound, is the main constituent in the roots and rhizomes of licorice (*Glycyrrhiza glabra*) and has anti-viral and anti-inflammatory effects. Several studies have shown that GL could attenuate the activation of TLRs and reduce macrophage cytokine release (Yang et al., 2010; Chang et al., 2014). This effect of GL could be a possible way to widen the therapeutic window of I/R injury (Zhang et al., 2014). However, few studies have been designed to investigate its use in a LIRI model.

The purpose of this study was to investigate the protective effects of GL in LIRI mice model and seek to explore the molecular mechanism responsible for its effects.

## Materials and Methods

### Animal Groups

The animal protocol was approved by the Institutional Animal Care and Use Committee of Guangxi Medical University (Nanning, China). All animal studies were carried out in accordance with the animal’s guidelines of the University Institutional Animal Care and Use Committee. Six-eight weeks male BALB/C mice (30 ± 2 g, Animal Centre, Guangxi Medical University, China) were randomly assigned to four groups: (1) Control group (not being exposed to anesthesia or surgery or any drugs), (2) Glycyrrhizin group (only injected intraperitoneally Glycyrrhizin), (3) I/R group (lung ischemia-reperfusion), (4) I/R+ Glycyrrhizin group.

### Glycyrrhizin Application

Glycyrrhizin acid ammonium salt (catalog number: sc-203059; Santa Cruz, CA, United States) was dissolved in acetic acid and injected intraperitoneally at 200 mg/kg at 30 min before anesthesia. The dose and usage of GL were chosen based on previous study (Kim et al., 2015).

### Animal Model of LIRI

Mice were anesthetized by intraperitoneal injection of 10% chloral hydrate (4.5 mL/kg). Next, mechanical ventilation was applied using a RSP1002-type small animal ventilator with a respiratory ratio (inspired to expired air) of 1:1 and a breathing frequency 80 breaths/min. The tidal volume was 10 mL/kg and the fraction of inspired oxygen was 100%. Lung collapse and expansion were observed. The mice in I/R group had underwent thoracotomy, occluded the left hilus pulmonis (including pulmonary artery, vein, and bronchi) by a microvascular clamp for 60 min, and followed by 120 min of reperfusion before closing thoracic incision (Mussi et al., 2008). At 2 h after operation, mice were sacrificed by cervical vertebra dislocation. Portions of the lower lobe of the left lung were excised from mice for measurement in all groups. No mouse mortality was recorded during the experimental approach.

### Hematoxylin and Eosin (H&E) Staining

As we described previously (Lin et al., 2015), mice lung tissues were fixed in 4% (w/v) paraformaldehyde and embedded in paraffin. Sections were prepared and evaluated by experienced pathologists who were blinded to the experimental treatment conditions. The thickness of the slices (4 microns) were waxed off by xylene and hydration, stained 5 min by hematoxylin, differentiated 30 s by hydrochloric acid ethanol, soaked 15 min in water, stained 2 min by eosin. And then the slices of H&E staining were completed after conventional dehydration, transparent and sealing.

### Microscopy and Histology Scoring of Lung Injury

Hematoxylin and Eosin stained paraffin-mounted lung sections were examined by light microscopy. An investigator who was blinded to the group assignment was assigned to examine the lung H&E slides and determine the levels of lung injury with a scoring system. For each mouse, 10 fields were examined at 200× total magnification. Scoring was performed as described elsewhere (Prakash et al., 2015). Briefly, the first criterion was infiltration or aggregation of inflammatory cells in air space or vessel walls: 1 = only wall, 2 = few cells in air space, 3 = intermediate, 4 = severe (air space congested). Second criterion was interstitial congestion and hyaline membrane formation: 1 = normal lung, 2 = moderate (>25% of lung section), 3 = intermediate (25–50% of lung section), 4 = severe (>50% of lung section). Third criterion was hemorrhage: 0 = absent, 1 = present.

### Determination of Wet/Dry Ratio

As we described previously (Dai et al., 2015), Pulmonary wet/dry ratios were measured as an index of pulmonary edema and congestion. After mice were killed, the lower lobe of the left lung was immediately weighed and then dried to a constant weight at 60°C for 24 h.

### Collection of BALF and Alveolar Macrophages

Alveolar macrophages were isolated as we described previously (Dai et al., 2015). In brief, the lungs were flushed once with 5 mL of cold phosphate buffered saline through the cannulated trachea (Dulbecco’s PBS; Gibco BRL, Grand Island, NY, United States) to collect bronchoalveolar lavage fluid (BALF). The lungs were subsequently flushed another eight times with 10 mL PBS to obtain alveolar macrophages.

### Pulmonary Vascular Permeability through Total Proteins in BALF

The level of total proteins in BALF is a marker of alveolar vascular permeability. Determination of total proteins was performed using the bicinchoninic acid (BCA) assay according to the manufacturer’s instructions (Pierce, Rockford, IL, United States).

### Counting of Alveolar Macrophages

As we described previously (Dai et al., 2015), BALF was resuspended in Dulbecco’s modified Eagle medium (DMEM, Gibco, United States), counted, and transferred to 24-well culture plates (BD, Franklin Lakes, NJ, United States). After incubation for 60 min at 37°C in a 5% CO_2_ atmosphere, cultures were washed with DMEM to remove non-adherent cells. The adherent cells were counted using a hemocytometer, viability was determined using a 0.2% trypan blue exclusion assay, and cell differentiation and aggregation were examined by counting 500 cells on a Wright-Giemsa-stained slide. These cultures also served as the source for analyzing protein expression in alveolar macrophages.

### Western Blot Analysis

The left lung tissues (50μg) and alveolar macrophages (5 × 10^6^) were homogenized in RIPA buffer (catalog number: 89900; Thermo Scientific, Worcester, MA, United States) containing protease inhibitor cocktail (catalog number: P2714; Sigma, St Louis, MO, United States) and phosphatase inhibitor cocktail (catalog number: 04906845001; Roche Applied Science, Indianapolis, IN, United States). Homogenates were centrifuged at 13,000 rpm at 4°C for 20 min. The supernatant was collected as the total proteins of lung tissues and alveolar macrophages. The protein concentration was determined by Pierce BCA protein assay kit (catalog number: 23227; Pierce Biotechnology, Rockford, IL, United States). Twenty microgram proteins per lane were separated on a polyacrylamide gel. The proteins were then transferred onto a polyvinylidene difluoride membrane. The membranes were incubated with the following primary antibodies overnight at 4°C: rabbit monoclonal anti-TLR2 antibody (1:1000, catalog number: ab108998; Abcam, Cambridge, MA, United States), anti-MyD88 antibody (1:1000, catalog number: ab2064; Abcam, Cambridge, MA, United States), anti-NF-κB p105/p50 antibody (1:1000, catalog number: ab32360; Abcam, Cambridge, MA, United States), mouse monoclonal anti-β-Actin antibody (1:5000, catalog number: ab6276; Abcam, Cambridge, MA, United States). Protein bands were visualized using enhanced chemiluminescence (Pierce, United States). The protein band intensities of TLR2, MyD88, NF-κB proteins were normalized to those of β-Actin. The results from animals under various experimental conditions then were normalized by mean values of the corresponding control animals.

### Lung Tissues and BALF Measurements of IL-1β and IL-6

ELISA kits for measuring mice IL-1β and IL-6 (catalog number: MLB00C and M6000B, respectively; R&D Systems, Minneapolis, MN, United States) were used to quantify the contents of these cytokines in the lung tissues and BALF according to the manufacturer’ instructions. The quantity of IL-1β and IL-6 in the lung tissues and BALF was standardized to the protein contents.

### Statistical Analysis

Parametric results in normal distribution are presented as mean ± SD (*n* ≥ 6). All data were analyzed by one-way analysis of variance followed by the Tukey test if the data were normally distributed or by one-way analysis of variance on ranks followed by the Tukey test if the data were not normally distributed. These non-normally distributed data were presented as box plots in the figures. Differences were considered significant at *P* < 0.05 based on two-tailed hypothesis testing. All statistical analyses were performed with SigmaStat (Systat Software, Point Richmond, CA, United States).

## Results

### GL Ameliorates LIRI in Morphology

**FIGURE 1:**
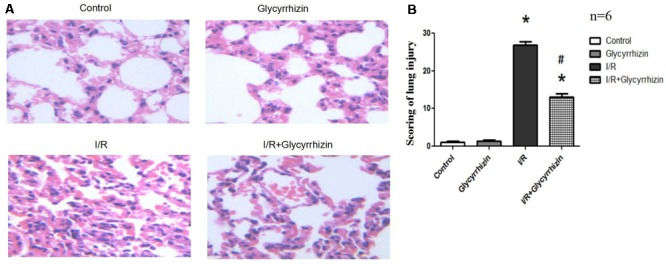
Morphological changes in Control, Glycyrrhizin, I/R and I/R+Glycyrrhizin groups by H&E staining (200×) **(A)**. The scoring of lung injury shown in **(B)** (^∗^*P* < 0.05, compared with control. ^#^*P* < 0.05, compared between I/R and I/R+Glycyrrhizin group).

Hematoxylin and Eosin staining and histology scoring of lung tissue were performed to assess the extent of lung injury. **Figure 1A** showed the results for lung morphology in all groups. In the control and GL groups, lung tissue structures were intact, lacking inflammatory cell infiltration and alveolar wall thickening. In I/R group, alveolar rupture and tissue congestion were observed, the alveolar septa had widened and ambiguous. In I/R+GL group, lung tissue structures were mainly intact, some neutrophils infiltration and red blood cells had leaked into the alveolar space. The lung injury score was significantly higher in I/R group compared with the control and GL group. The levels of lung I/R injury were significantly ameliorated in I/R+GL group compared with I/R group. No difference was noted between control group and GL group (**Figure 1B**).

### GL Decreases I/R-Induced Pulmonary Permeability and Edema

**FIGURE 2:**
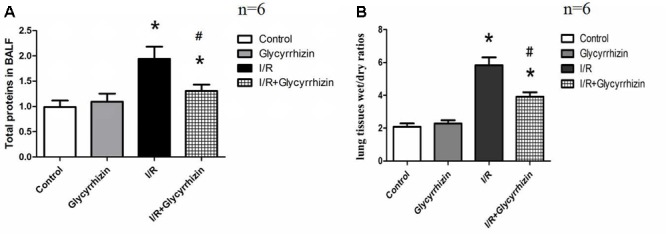
Glycyrrhizin decreases I/R-induced pulmonary permeability and edema. **(A)** Graphic presentation of Total proteins in BALF. **(B)** Graphic presentation of the Wet/dry ratios in lung tissues. (^∗^*P* < 0.05, compared with control. ^#^*P* < 0.05, compared between I/R and I/R+Glycyrrhizin group).

Pulmonary wet/dry ratios and the levels of total proteins in BALF were measured to assess pulmonary permeability and edema. **Figure 2** presented the levels of total proteins in BALF (**Figure 2A**) and the wet/dry ratios in lung tissues (**Figure 2B**). GL significantly reduced I/R-induced increases in the levels of total proteins in BALF (*P* < 0.05) and the wet/dry ratios in lung tissue (*P* < 0.05).

### GL Reduces I/R-Induced Isolated Alveolar Macrophages

**FIGURE 3:**
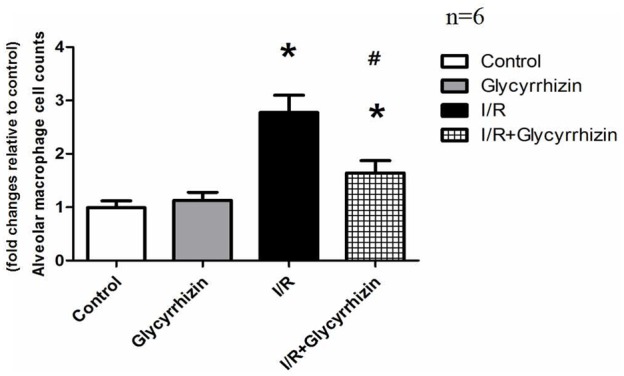
Isolated alveolar macrophage cell counts in BALF. (^∗^*P* < 0.05, compared with control. ^#^*P* < 0.05, compared between I/R and I/R+Glycyrrhizin group).

Isolated alveolar macrophages were counted to assess the activity of the alveolar macrophages. The percentage of alveolar macrophages was more than 90% counted by the hemocytometer. Compared with control and GL group, isolated alveolar macrophages in BALF had significantly increased in the I/R group (**Figure 3**). GL significantly reduced I/R-induced isolated alveolar macrophages.

### GL Inhibits I/R-Induced Inflammation in Lung Tissues and BALF

**FIGURE 4:**
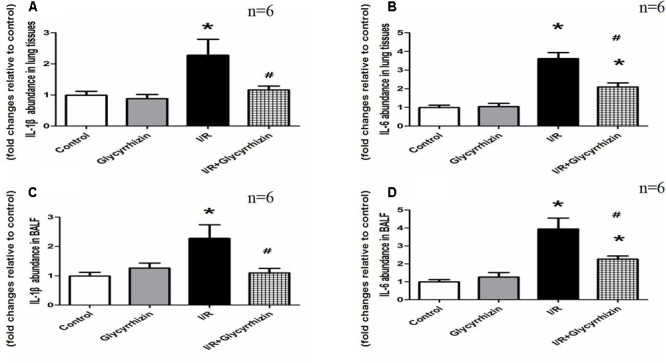
Glycyrrhizin inhibits I/R-induced IL-1β and IL-6 in lung tissues and BALF. **(A)** Graphic presentation of IL-1β abundance in lung tissues. **(B)** Graphic presentation of IL-6 abundance in lung tissues. **(C)** Graphic presentation of IL-1β abundance in BALF. **(D)** Graphic presentation of IL-6 abundance in BALF. (^∗^*P* < 0.05, compared with control. ^#^*P* < 0.05, compared between I/R and I/R+Glycyrrhizin group).

IL-1β and IL-6 in lung tissues and BALF were measured to assess the inflammatory response. **Figure 4** showed the levels of IL-1β and IL-6 in lung tissues and BALF. The results demonstrated that I/R injury significantly increased the levels of IL-1β and IL-6 in Lung tissues and BALF compared with the control and GL group (*P* < 0.05). GL significantly inhibited the I/R-induced IL-1β and IL-6 expression compared with the I/R group (*P* < 0.05).

### GL Inhibits I/R-Induced TLR2 Expression in Lung Tissues and Alveolar Macrophages

**FIGURE 5:**
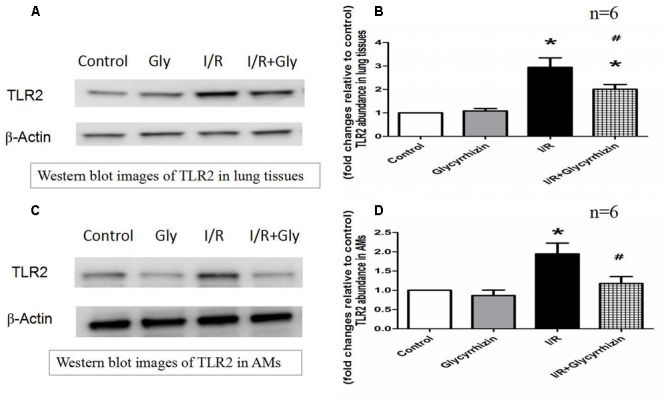
Glycyrrhizin inhibits I/R-induced TLR2 expression in Lung tissues and alveolar macrophages. **(A)** Representative Western blot images of TLR2 in lung tissues. **(B)** Graphic presentation of TLR2 abundance in lung tissues. **(C)** Representative Western blot images of TLR2 in AMs. **(D)** Graphic presentation of TLR2 abundance in AMs. (^∗^*P* < 0.05, compared with control. ^#^*P* < 0.05, compared between I/R and I/R+Glycyrrhizin group).

Toll-like receptor2 protein expression in Lung tissues and alveolar macrophages were determined by Western Blot Analysis. The results in **Figure 5** demonstrated that I/R injury had significantly increased the expression of TLR2 in lung tissues and alveolar macrophages compared with the control and GL group (*P* < 0.05). GL significantly inhibited the I/R-induced TLR2 expression compared with the I/R group(*P* < 0.05).

### GL Modulates the Expression of Downstream Factors of TLRs in Alveolar Macrophages

**FIGURE 6:**
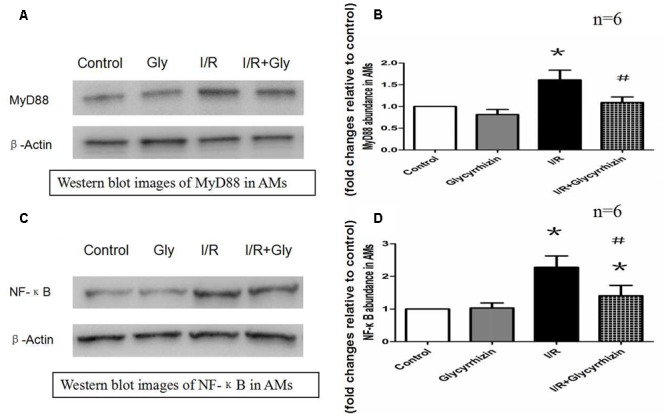
Glycyrrhizin inhibits I/R-induced MyD88 and NF-κB expression in AMs. **(A)** Representative Western blot images of MyD88 in AMs. **(B)** Graphic presentation of MyD88 abundance in AMs. **(C)** Representative Western blot images of NF-κB in AMs. **(D)** Graphic presentation of NF-κB abundance in AMs. (^∗^*P* < 0.05, compared with control. ^#^*P* < 0.05, compared between I/R and I/R+Glycyrrhizin group).

MyD88 and NF-κB protein expression in alveolar macrophages were determined to assess the expression of downstream factors of TLRs. The results in **Figure 6** demonstrated that I/R injury significantly increased the expression of MyD88 and NF-κB in alveolar macrophages compared with the control and GL group (*P* < 0.05). The results also illustrated that treatment with GL significantly reduced MyD88 and NF-κB expression compared with the I/R group.

## Discussion

This study showed that treatment with GL had a strong protective effect in the I/R lung. GL treatment was found to decrease I/R-induced pulmonary permeability and edema and ameliorate the levels of lung I/R injury in morphology. In addition, administration of GL significantly inhibited I/R-induced inflammation and isolated alveolar macrophages. The present results suggest that protective effect of GL in I/R is, at least in part, attributable to its inhibitory effects on TLR2 and subsequently inhibition of TLR2 downstream factors (MyD88 and NF-κB) in alveolar macrophages.

In this study, we chose BALB/c mouse for the animal model because BALB/c mouse had been widely used in animal experiment of immunology and physiology. Recently, a number of studies have indicated that I/R injury is characterized by acute local inflammation. During reperfusion after acute ischemia, neutrophils are believed to exacerbate tissue damage by obstruction of vessels, augment of pulmonary permeability and release of proinflammatory cytokines and cytolytic enzymes. However, Why relatively short interruption and reperfusion of blood flow lead to inflammation remains an unresolved question of human physiology. And how to control the process of I/R inflammatory response is still an important issue to exploration.

Toll-like receptors refer to biomolecules produced by invading microbes or released from damaged tissue. These biomolecules serve as danger signals that initiate an inflammatory immune response. TLR2 is crucial for PAMP signaling and can work through the adaptor protein MyD88, which activates NF-κB and ultimately stimulates the production of proinflammatory cytokines (Shigeoka et al., 2007; Yang et al., 2010). TLRs trigger inflammation mediated by complement, macrophages, and neutrophils. These cells produce chemokines or cytokines that mediate systemic immune responses and recruit leukocytes to the sites of inflammation (Miyake, 2007; Ding et al., 2017). In the process of systemic immune reaction following exposure to harmful stimulus, alveolar macrophages are the primary producers of proinflammatory cytokines in lungs (Wang et al., 2012). The rapidly activated of alveolar macrophages suggests that they may play a crucial role in the pathogenesis of lung injury. Several previous reports have demonstrated removing alveolar macrophages from rats or mice could attenuate the alveolar barrier dysfunction and inflammatory lung injury (Frank et al., 2006; Eyal et al., 2007).

In our previous study (Dai et al., 2015; Huang et al., 2017), the results indicated that TLR2, TLR4, and TLR9 on alveolar macrophages and release of pro-inflammatory cytokines play a role in ventilator-induced lung injury. In this study, expression of TLR-2 and its downstream factors (MyD88 and NF-κB) in alveolar macrophages were significantly enhanced in I/R group compared to control group. These results are in accordance with previous other study (Ma et al., 2013) who demonstrated an upregulation of TLR-2, TLR-4, and MyD88 in MCAO stroke model. Moreover, TLR-2 deficiency caused significant reduction of infarct volume and suppressed inflammatory cytokine expression in infiltrating macrophages after brain ischemia (Shichita et al., 2012). Therefore, we believe that alveolar macrophages are rapidly activated, and the expression of TLR2 in alveolar macrophages plays an important role in the initiation of inflammation during the process of lung I/R.

Now the effective and applicable pharmacological treatments for LIRI is still absent. GL is a natural triterpene glycoside that has multiple biological activities, including anti-inflammatory, anti-viral, anti-oxidative, anti-cancerous, immune adjustment, organ-protective activities (Kim et al., 2011; Gong et al., 2012). The organ-protective effects of GL were previously evidenced by HMGB1-TLR4-IL-17A signaling pathway in the postischemic brain (Zhang et al., 2014), inhibition of reactive oxygen spices generation by neutrophils (Asl and Hosseinzadeh, 2008), ameliorating ischemic brain damage through downregulation of the TLR signaling cascade (Barakat et al., 2014). Previously reports also proved that GL has anti-inflammatory effects. In kainic acid-induced seizure animal model, treatment with GL might be attributable to the inhibitions of HMGB1 induction and release, which in turn, mitigates the inflammatory process (Luo et al., 2014). In rat skin thermal injury model, GL might possesses an anti-inflammation effect to protect the remote organs from burn-induced injury (Shen et al., 2015). All these actions make GL might be a suitable candidate for the treatment of LIRI. However, few studies have been designed to investigate its use in a LIRI model. In the current study, GL significantly reduced the lung injury score, decreased I/R-induced pulmonary permeability and isolated alveolar macrophages, inhibited I/R-induced inflammation in lung tissues and BALF. Our results indicated that treatment with GL have anti-inflammatory and organ-protective effects in LIRI animal model.

The mechanism by which GL exerted these effects is still unclear. Kim considered that GL had inhibitory effect on oxidative damage and suppress caspase-3 activation (Kim and Lee, 2008). Ogiku et al. (2011) found that GL protected ischemia reperfusion liver injury by stabilization of the membrane structure of hepatocytes. In addition, GL was reported to attenuate inflammatory responses induced by TLR-3 and TLR-4, alter the integrity of the plasma membrane and attenuate receptor mediated signaling (Schröfelbauer et al., 2009). Barakat et al. (2014) found that GL suppressed the expression of TLR-2, TLR-4 expression in brain ischemia. In the present study, our data indicated that treatment with GL suppressed I/R-induced TLR2 expression in Lung tissues and alveolar macrophages and modulated the expression of downstream factors of TLR2. Our results are in accordance with previously reports. Moreover, to our knowledge, this is the first study to indicate the effect of GL on the expression of TLR2 and its downstream factors in alveolar macrophages after LIRI. This could be attributed to a reduction in alveolar macrophages activation with the resultant decrease TLR2 and inflammatory mediators.

## Conclusion

Pretreatment with GL could ameliorate I/R lung injury. The mechanisms of its protective effects might be inhibit I/R-induced inflammatory response through downregulate TLR2 signaling cascade in alveolar macrophages.

## Author Contributions

LF: Study design, fund collection, samples detection, manuscript preparation. FJ: Data interpretation, samples detection, literature search. WT: Data collection, animal model. HY: Statistical analysis. PL: Study design, fund collection, manuscript preparation.

## Conflict of Interest Statement

The authors declare that the research was conducted in the absence of any commercial or financial relationships that could be construed as a potential conflict of interest.
